# Supplementary material for: Genomic investigation reveals local persistence and regional circulation of dengue virus associated with recent outbreaks in Kenya
Source: Sci Rep. 2026 Jul 3;16:30608. doi: 10.1038/s41598-026-59068-8 (PMC13630545; doi:10.1038/s41598-026-59068-8)
Supplement: Supplementary file 1 [file 41598_2026_59068_MOESM1_ESM.pdf]

Genomic investigation reveals local persistence and regional circulation of dengue virus associated with recent outbreaks in Kenya

Solomon K. Langat[1,4,#], Sheila Kageha[1,4], Victor Jeza[2], Genay Pilarowski[3], Albert Nyunja[1], Paul E. Oluniyi[3], Juliana Gil[3], Nelly Ogada[2], Lewis Gande[2], Jane Thiiru[1], Seth Okeyo[1], Pascah Bulia[1], Jael Amugongo[2], Victor Ofula[1], Francis Mutuku[2], Cristina M. Tato[3], Samoel Khamadi[1], Joel Lutomiah[1], Konongoi Limbaso[1].

1. Centre for Virus Research, KEMRI, Nairobi, Kenya
2. Technical University of Mombasa, Mombasa, Kenya
3. Chan Zuckerberg Biohub, San Francisco, USA
4. These authors contributed equally to this work

\# Correspondence: lsolomon@kemri.go.ke

Supplementary

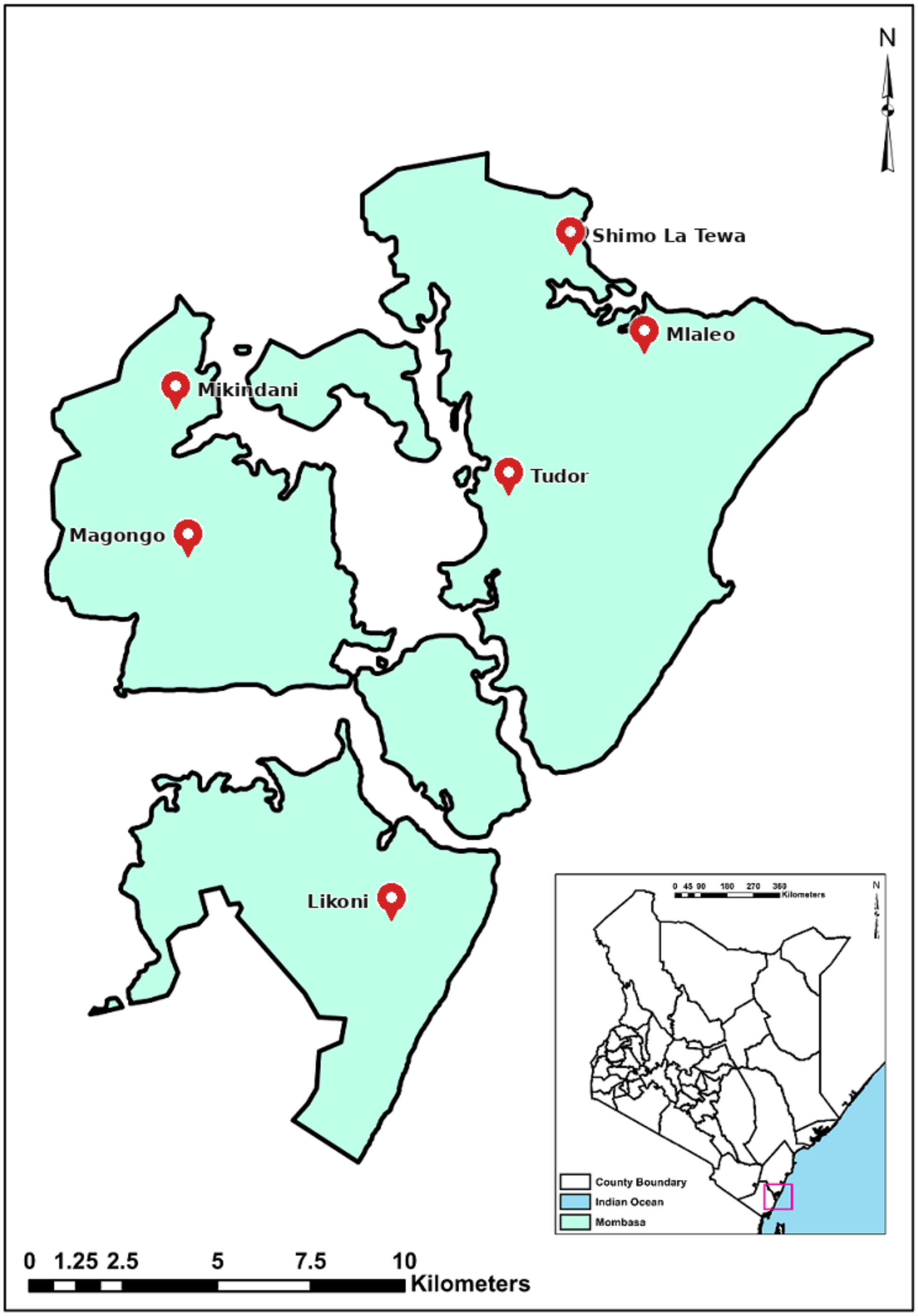

Figure S1. Map of Mombasa County showing the geographic locations of samples included in the 2023 dengue outbreak investigation. Inset: Map of Kenya indicating the location of Mombasa County. The map was created for this study by Samuel Owaka using ArcGIS Desktop version 10.2.2 (Esri, Redlands, CA, USA; http://desktop.arcgis.com/en/arcmap). Administrative boundary data for Kenya were obtained from the Global Administrative Areas (GADM) database, version 4.1 (https://gadm.org).

Table S1: Overview of sample sources and datasets used in this study.

Dataset role	Sample source	Collection period	Location	Study type	Ethical approval	Consent procedure
**Primary study dataset**	2023 dengue outbreak investigation	2023	Mombasa County, Kenya	Outbreak investigation	KEMRI-SERU SSC #4499	Written informed consent obtained from adult participants; parental/guardian consent obtained for participants <18 years
**Contextual dataset**	KEMRI/DDSR arbovirus surveillance	2020–2022	Multiple counties, Kenya	Routine arbovirus surveillance	KEMRI-SERU SSC #3035	Waiver of individual consent for anonymized residual diagnostic specimens collected during public health response activities
**Contextual dataset**	TUM febrile illness surveillance	2020–2022	Mombasa and Kwale Counties, Kenya	Prospective febrile illness surveillance	TUM ERC EXT/005/2022	Written informed consent obtained from adult participants; parental/guardian consent obtained for participants <18 years
**Post-outbreak contextual dataset**	TUM febrile illness surveillance	2024–2025	Mombasa and Kwale Counties, Kenya	Prospective febrile illness surveillance	TUM ERC EXT/005/2022	Written informed consent obtained from adult participants; parental/guardian consent obtained for participants <18 years

Table S2: Sample metadata and molecular characteristics of dengue virus sequences generated in the study

Sample Information			Molecular Characteristics			
Strain	County	Collection Date	Sequence Length	Serotype	Genotype	Genbank Accession
TUM_F17	Mombasa	16-Jan-25	10653	DENV-1	3	PV717359
RR175008	Kwale	Aug-20	10667	DENV-1	3	PV717359
RR175025	Kwale	Jun-21	10667	DENV-1	3	PV717360
RR175004	Kwale	Jan-20	10667	DENV-1	3	PV717361
LIK_055	Mombasa	12-Jun-23	10653	DENV-1	3	PV717362
MIK_010	Mombasa	12-Apr-23	10667	DENV-1	3	PV717363
MIK_020	Mombasa	24-May-23	10651	DENV-1	3	PV717364
SLT_048	Mombasa	03-Jul-23	10580	DENV-1	3	PV717365
LIK_016	Mombasa	14-Mar-23	4554	DENV-1	3	PV717366
MAG_067	Mombasa	13-Jun-23	10590	DENV-1	3	PV717367
MAG_036	Mombasa	15-Jun-23	10257	DENV-1	3	PV717368
MLA_033	Mombasa	02-May-23	10645	DENV-1	3	PV717369
MIK_004	Mombasa	14-Mar-23	10667	DENV-1	3	PV717370
MIK_017	Mombasa	24-May-23	10655	DENV-1	3	PV717371
RR176_0110222	Lamu	01-Feb-22	10667	DENV-1	3	PV717372
RR176_0120222	Lamu	01-Feb-22	10667	DENV-1	3	PV717373
RR176_0270222	Lamu	09-Feb-22	10667	DENV-1	3	PV717374
RR176_0380222	Lamu	09-Feb-22	10667	DENV-1	3	PV717375
RR176_0430222	Lamu	09-Feb-22	10667	DENV-1	3	PV717376
RR176_0460222	Lamu	19-Feb-22	10660	DENV-1	3	PV717377
RR176_0480222	Lamu	19-Feb-22	9807	DENV-1	3	PV717378
MSA_0110124	Mombasa	Jan-24	10176	DENV-2	2	PV717379
MSA_0050124	Mombasa	Jan-24	10659	DENV-2	2	PV717380
RR175005	Kwale	Aug-21	10659	DENV-2	2	PV717381
LIK_059	Mombasa	14-Jun-23	7073	DENV-2	2	PV717382
MLA_014	Mombasa	14-Apr-23	10659	DENV-2	2	PV717383
MLA_039	Mombasa	06-Jul-23	10557	DENV-2	2	PV717384
MLA_040	Mombasa	11-Jul-23	10659	DENV-2	2	PV717385
MLA_065	Mombasa	30-Jun-23	7743	DENV-2	2	PV717386
MLA_067	Mombasa	03-Jul-23	10659	DENV-2	2	PV717387
SLT_041	Mombasa	23-Jun-23	10659	DENV-2	2	PV717388
SLT_046	Mombasa	03-Jul-23	10659	DENV-2	2	PV717389
TUD_036	Mombasa	17-Jun-23	10659	DENV-2	2	PV717390
TUD_050	Mombasa	19-Jun-23	10615	DENV-2	2	PV717391
TUD_051	Mombasa	20-Jun-23	10659	DENV-2	2	PV717392

TUD_041	Mombasa	21-Jun-23	10560	DENV-3	3	PV717393
MAG_066	Mombasa	13-Jun-23	10467	DENV-3	3	PV717394
MIK_032	Mombasa	18-Apr-23	10121	DENV-3	3	PV717395
