# Supplementary material for: Genomic investigation reveals local persistence and regional circulation of dengue virus associated with recent outbreaks in Kenya
Source: Sci Rep. 2026 Jul 3;16:30608. doi: 10.1038/s41598-026-59068-8 (PMC13630545; doi:10.1038/s41598-026-59068-8)
Supplement: Supplementary file 2 [file 41598_2026_59068_MOESM2_ESM.pdf]

Supplementary Appendix

All genome sequences and associated metadata supporting the findings of this study can be accessed through the persistent digital object identifier https://doi.org/10.55876/gis8.260526ra

In addition to the minted DOI, GISAID also communicates the aggregation of GISAID accession numbers (EPI_ISL_IDs) through the corresponding EPI_SET_260526ra identifier to facilitate both, the acknowledgment of all data contributors and the direct retrieval of the underlying data from GISAID used in this study.

Dengue Virus Data Summary

GISAID Identifier	Digital Object Identifier	Number of individual viruses	Data Collection range	Number of countries/territories
EPI_SET_260526ra	https://doi.org/10.55876/gis8.260526ra	1,083	2022-01-11 to 2025-01-31	49
